# COVID-19 as a Complex Intergovernmental Problem

**DOI:** 10.1017/S0008423920000281

**Published:** 2020-04-14

**Authors:** Mireille Paquet, Robert Schertzer

**Affiliations:** 1 Department of Political Science, Concordia University, Montreal, QC H3G 1M8; 2 Department of Political Science, University of Toronto, Toronto, ON M5S 3G3

## Abstract

Federations increasingly face complex policy challenges, from managing climate change to mass migration. COVID-19 is a prime example of this emerging type of problem. This research note introduces the concept of *complex intergovernmental problems* (CIPs) to better understand these types of challenges.

Federations increasingly face complex policy challenges, from managing climate change to mass migration. COVID-19 is a prime example of this emerging type of problem. This research note introduces the concept of *complex intergovernmental problems* (CIPs) to better understand these types of challenges.

While political leaders and media often describe COVID-19 as a crisis, the concept of CIPs generates more analytical power to understand the management of this pandemic in federations and multilevel governance systems. The nature of this problem requires intergovernmental coordination and cooperation for effective policy responses. At the same time, COVID-19 will significantly affect intergovernmental relations in Canada over both the short and long terms. Highlighting how COVID-19 intersects with intergovernmental relations allows us to better assess how governments have responded and will facilitate comparative research.

## Complex Intergovernmental Problems

Complex intergovernmental problems (CIPs) are boundary-spanning, irreducible policy problems that unfold within an intergovernmental system (see Thomann et al., 2019; Versluis et al., 2019). This concept draws upon studies of intergovernmental relations in Canada, particularly work on the factors influencing collaboration (Cameron and Simeon, 2002; Skogstad and Bakvis, 2012). It also builds upon insights from public policy and multilevel governance research (see, for example, Maggetti and Trein, 2019; Irepoglu Carreras, 2019; Paquet, 2017). Instead of taking federal arrangements as a starting point, this approach focusses on the nature and characteristics of a policy problem to analyze how governance systems and actors adapt. Public policy scholars have long studied how the social construction and the nature of different policy problems affect politics, policy designs and policy outcomes (see, for example, Peters, 2017; Béland, 2009; Rochefort and Cobb, 1993). They have developed typologies of problem attributes and different concepts to account for varying degrees of problem complexity (Head, 2019). For example, problems that are multicausal and highly interdependent are sometimes characterized as “wicked problems” (Peters, 2017).

We define CIPs as distinct from wicked problems or crises because of their inherent intergovernmental nature and related consequences. CIPs—such as the opioid crisis, pandemics or climate change—have three characteristics. First, addressing their root causes is not something that is amenable to resolution by the actions of any one government. Instead, governments can generally only address the consequences of the problem on their territory and within their regulatory space. Second, the nature of CIPs requires high levels of coordination and collaboration among implicated governments. Responding to their consequences cannot be achieved by a single actor in an intergovernmental system. Third, these problems challenge the existing norms and venues of intergovernmental relations. Often, the novelty of a CIP requires close collaboration from agencies and governments that have not traditionally worked together. They can also create situations where joint interventions are necessary even though the existing mandates, agendas and processes of intergovernmental forums are not well suited to coordinate government action. Similarly, a novel CIP can exacerbate poorly functioning aspects of intergovernmental relations—representing a stress test that exposes cracks in the system. Failure to effectively respond to CIPs can also have trickle-down effects on intergovernmental relations in other sectors, including conflicts or disengagement.

In short: CIPs generate pressure to act in novel ways and to establish new forms of collaboration, which can be difficult even under ideal conditions. These problems create barriers to collaboration because they call into question the existing power equilibriums and dominant narratives about how to work together and share responsibilities within intergovernmental systems. CIPs are thus somewhat paradoxical: they demand intergovernmental collaboration for effective and legitimate policy responses, while making the necessary collaboration difficult to achieve.

## COVID-19 as a CIP

In Canada and other federations, COVID-19 aligns with all the attributes of a CIP. The global spread of the virus has reached a point where government actions are now focussed on managing its consequences. However, mitigation measures cannot be implemented unilaterally by any one government.

Within Canada, the response to COVID-19 requires and challenges the intergovernmental system. The need for intergovernmental collaboration is most acute in the public health sector. This necessity reflects the provincial responsibility for healthcare, paired with a significant role for the federal government in financing the system and leading the pandemic response through the Public Health Agency of Canada. This federal role is a clear legacy of the 2003 SARS outbreak (Wilson and Lazar, 2005). The main policy responses to COVID-19 have been to implement social distancing measures and to ensure the healthcare system has the resources it needs to treat patients. Decisions on social distancing measures are derived from information and statistical models shared across jurisdictions. The sharing of resources such as testing kits, masks and ventilators across the country will increasingly be a key determinant of effective responses (Chouinard, 2020). It is impossible for Ottawa to address COVID-19 unilaterally, even if it were to implement its sweeping emergency powers (Swiffen, 2016). At the same time, the pandemic demonstrates that provinces and territories are dependent on the decisions and capacities of other governments—at all levels—to continue to act within their own regulatory space.

But the scope of COVID-19 is so profound that it engages many other aspects of Canada's intergovernmental system. Indeed, the consequences of COVID-19 extend beyond public health. In the short term, they include mobility control and international and interprovincial trade and supply chains, as well as the provision of basic income security measures for Canadians. In the medium term, governments will need to find creative ways to address the economic impact of the pandemic on their revenues and budgets. Given the increased spending not only on healthcare but also on other social support measures, these costs will be particularly felt among provinces and municipalities. None of these policy challenges can be addressed by a single order of government. At the same time, the existing intergovernmental processes and norms of working together in Canada may pose barriers to this needed collaboration. For instance, the public health sector has eschewed the establishment of dedicated intergovernmental venues. It has favoured ad hoc mechanisms to share specialized knowledge and lacks a legacy of first ministers working closely together and trusting each other on the issue. Beyond public health, the dramatic nature of COVID-19's impact on our society and economy will represent a stress test for existing peak and sectoral intergovernmental venues, especially considering pre-COVID-19 conflicts within the federation.

## Intergovernmental Relations and CIPs

What are the advantages of labelling COVID-19 as a CIP? Besides providing a rich descriptive framework, the concept allows us to focus on the inherent intergovernmental nature of the crisis. Applying this lens helps to illuminate two sides of the impact of a CIP like COVID-19. On one side is how the intergovernmental system impacts the effective management of the problem's consequences. Understanding how the structure, norms, relationships and processes of the intergovernmental system are helping or hindering the response to the crisis is central to explaining policy outputs and the outcomes associated with COVID-19. How governments in Canada work together—or do not—to share critical medical supplies will impact health outcomes. Similarly, how governments coordinate to access global capital and credit to shore up strained budgets will shape their ability to continue to provide essential services to Canadians.

On the other side, CIPs are likely to significantly impact intergovernmental relations over the short, medium and long terms. In the case of COVID-19, these effects could range from the creation of new venues for federal-provincial-territorial (FPT) collaboration in public health and emergency preparedness, to a complete rearrangement of power dynamics between Ottawa and the provinces in the face of a long global economic crisis in which the federal spending power will be an important mechanism. Our research into a previous CIP in Canada—irregular border crossings—demonstrated a clear pattern of the short-term, medium-term and long-term effects that these types of problems can have on the intergovernmental system (Schertzer and Paquet, 2020). In the immediate face of a surge of irregular border crossings in 2017, governments and public servants rallied and collaborated to ensure that government operations remained effective. They provided innovative responses to the new realities on the ground. In the medium and long term, however, significant political conflicts over fiscal federalism and distribution emerged—tensions that are still not resolved. One of the key takeaways from the surge in irregular border crossings in Canada is that a CIP evolves over time: initial periods of crisis management and collaboration can give way to intergovernmental conflict. Often, these conflicts can be exacerbated when the policy issue itself gains political salience. So, while there are reasons to celebrate current FPT collaboration in response to COVID-19, over time, competing interests, resource constraints, legacies from past conflicts, and weak points in the intergovernmental system are likely to create significant tension.

Beyond Canada, the concept of CIPs can be used to compare responses to, and the consequences of, COVID-19 in other federations. In the United States, Australia, India and Germany—to name only a few examples—the pandemic response is clearly being shaped by intergovernmental relations. Comparative analysis will help to document how different intergovernmental systems respond to COVID-19, along with the impact of particular institutional and political variables. Furthermore, comparison can identify factors that contribute to different degrees of adaptability of intergovernmental structures and norms, while also tracing over time how conflicts (for example, related to fiscal federalism) become embedded into the management of this CIP. Likewise, our approach opens the door to fruitful within-case comparisons (for example, multiple CIPs in a given federation) and cross-case comparisons of other CIPs.

Understanding how intergovernmental systems are influencing policy responses to COVID-19 is critical. At the same time, understanding how COVID-19 is impacting intergovernmental systems in federations is also essential. Seeing COVID-19 as a CIP can help us in both research endeavours.

## References

[ref1] Béland, Daniel. 2009 “Ideas, Institutions, and Policy Change.” Journal of European Public Policy 16 (5): 701–18.

[ref2] Cameron, David and Richard Simeon. 2002 “Intergovernmental Relations in Canada: The Emergence of Collaborative Federalism.” Publius: The Journal of Federalism 32 (2): 49–72.

[ref3] Chouinard, Stéphanie. 2020 “COVID-19 Crisis Sheds Light on Blind Spot of Canadian Federalism: Interprovincial Collaboration.” *iPOLITICS*, April 9. https://ipolitics.ca/2020/04/09/covid-19-crisis-sheds-light-on-blind-spot-of-canadian-federalism-interprovincial-collaboration/.

[ref4] Head, Brian W. 2019 “Forty Years of Wicked Problems Literature: Forging Closer Links to Policy Studies.” Policy and Society 38 (2): 180–97.

[ref5] Irepoglu Carreras, Yasemin. 2019 “Problem-Solving across Literatures: Comparative Federalism and Multi-Level Governance in Climate Change Action.” European Policy Analysis 5 (1): 117–34.

[ref6] Maggetti, Martino and Philipp Trein. 2019 “Multilevel Governance and Problem-Solving: Towards a Dynamic Theory of Multilevel Policy-Making?” Public Administration 97 (2): 355–69.

[ref7] Paquet, Mireille. 2017 “Wicked Problem Definition and Gradual Institutional Change: Federalism and Immigration in Canada and Australia.” Policy and Society 36 (3): 446–63.

[ref8] Peters, Guy. 2017 “What Is So Wicked about Wicked Problems? A Conceptual Analysis and a Research Program.” Policy and Society 36 (3): 385–96.

[ref9] Rochefort, David A. and Roger W. Cobb. 1993 “Problem Definition, Agenda Access, and Policy Choice.” Policy Studies Journal 21 (1): 56–71.

[ref10] Schertzer, Robert and Mireille Paquet. 2020 “How Well Is Canada's Intergovernmental System Handling the Crisis?” *Policy Options*, April 8. https://policyoptions.irpp.org/magazines/april-2020/how-well-is-canadas-intergovernmental-system-handling-the-crisis/.

[ref11] Skogstad, Grace and Herman Bakvis. 2012 “Conclusion: Taking Stock of Canadian Federalism” In Canadian Federalism: Performance, Effectiveness and Legitimacy, ed. Herman Bakvis and Grace Skogstad. Toronto: Oxford University Press.

[ref12] Swiffen, Amy. 2016 “The Hermeneutics of Jurisdiction in a Public Health Emergency in Canada.” International Journal for the Semiotics of Law/Revue internationale de Sémiotique juridique 29 (3): 667–84.

[ref13] Thomann, Eva, Philipp Trein and Martino Maggetti. 2019 “What's the Problem? Multilevel Governance and Problem-Solving.” European Policy Analysis 5 (1): 37–57.

[ref14] Versluis, Esther, Marjolein van Asselt and Jinhee Kim. 2019 “The Multilevel Regulation of Complex Policy Problems: Uncertainty and the Swine Flu Pandemic.” European Policy Analysis 5 (1): 80–98. 10.1002/epa2.1064 PMC7163688 34616904

[ref15] Wilson, Kumanan and Harvey Lazar. 2005 “Planning for the Next Pandemic Threat: Defining the Federal Role in Public Health Emergencies.” IRRP Policy Matters 6 (5): 1–36.

